# Comparing ∆T_max_ Determination Approaches for Granier-Based Sapflow Estimations

**DOI:** 10.3390/s16122042

**Published:** 2016-12-01

**Authors:** Inken Rabbel, Bernd Diekkrüger, Holm Voigt, Burkhard Neuwirth

**Affiliations:** 1 Department of Geography, University of Bonn, Meckenheimer Allee 166, 53115 Bonn, Germany; b.diekkrueger@uni-bonn.de; 2 Center for Development Research, University of Bonn, Walter-Flex-Straße 3, 53113 Bonn, Germany; hvoigt@uni-bonn.de; 3 Dendro-Labor Windeck, Tree-Ring Analytics, 51570 Windeck, Germany; b.neuwirth@online.de

**Keywords:** heat dissipation, thermal dissipation, maximum temperature gradient, data processing, transpiration, Norway spruce, *Picea abies*

## Abstract

Granier-type thermal dissipation probes are common instruments for quantifying tree water use in forest hydrological studies. Estimating sapflow using Granier-type sapflow sensors requires determining the maximum temperature gradient (∆T_max_) between the heated probe and the reference probe below. ∆T_max_ represents a state of zero sap flux, which was originally assumed to occur each night leading to a ∆T_max_ determination on a daily basis. However, researchers have proven that, under certain conditions, sapflow may continue throughout the night. Therefore alternative approaches to determining ∆T_max_ have been developed. Multiple ∆T_max_ approaches are now in use; however, sapflow estimates remain imprecise because the empirical equation that transfers the raw temperature signal (∆T) to sap flux density (F_d_) is strongly sensitive to ∆T_max_. In this study, we analyze the effects of different ∆T_max_ determination approaches on sub-daily, daily and (intra-)seasonal F_d_ estimations. On this basis, we quantify the uncertainty of sapflow calculations, which is related to the raw signal processing. We show that the ∆T_max_ determination procedure has a major influence on absolute ∆T_max_ values and the respective sap flux density computations. Consequently, the choice of the ∆T_max_ determination approach may be a significant source of uncertainty in sapflow estimations.

## 1. Introduction

Granier-type thermal dissipation probes [1] are common instruments for quantifying tree water use in forest hydrological studies [2,3,4,5,6]. Upscaled to a ground area basis, tree water use is particularly valuable in accounting for actual tree transpiration when partitioning forest evapotranspiration [7,8,9]. The Granier system consists of two sensor probes inserted radially into the sapwood, one above the other. The upper probe is equipped with a heating element and a thermocouple, thus recording the heat dissipation due to sapflow. The lower probe measures the ambient reference temperature of the wood [10]. Sap flux density derived from the temperature gradient between the two probes using the empirical equation [1,11]:

(1) $$F_{d}=119\times {\left(\frac{\Delta T_{\max}-\Delta T}{\Delta T}\right)}^{1.231}$$

where F_d_ is the sap flux density (g·m^−2^·s^−1^), ∆T is the actual temperature gradient between the two probes and ∆T_max_ the maximum temperature gradient measured between the probes in a given time period.

Granier’s formula is strongly sensitive to the parameter ∆T_max_, which represents a state of zero sapflow (F_d_ = 0). Such zero flow conditions were originally assumed to occur every night [1,11]. This assumption led to a ∆T_max_ determination on a daily basis (D). However, there is increased evidence that, under certain conditions, sapflow continues throughout the night [12,13,14]. To improve nocturnal sapflow detection, researchers pay much attention to determining the zero flow conditions and respective ∆T_max_ values. Consequently, alternative approaches to determining ∆T_max_ have been developed, all based on the assumption that zero flow is related to erratically occurring ambient conditions.

There are two main approaches to including this assumption into sapflow calculations: (1) presuming the recurrence of zero flow within a given time period, during which ∆T_max_ is determined (empirical approaches) or (2) defining ∆T_max_ when accompanying environmental measures suggest that presumed zero flow conditions have been met (physically based approaches). Most commonly used are the empirical moving window approaches (MW), where ∆T_max_ is determined within dynamic time windows of different widths. Within these time windows, zero flux is assumed to occur only once. While Lu et al. [2] proposed estimating ∆T_max_ over periods of 7–10 days, in practice researchers applied moving windows of 3 days [8], 4- to 5-days [15], 7 days [16,17], 10 days [18,19,20] or even 14-day MWs [21] for ∆T_max_ determination. The subjectivity of selecting the MW width has already been identified as a drawback of this approach [22]. The advantage of MW approaches is that they are easy to implement and, due to their dynamic character, capable of compensating drifts in the data.

Another empirical procedure to overcome both the drift phenomena and the problem of nocturnal flow detection is performing a linear regression (LR) of ∆T_max_ values that have first been determined by a 10-day moving window [2,11]. This approach has been refined by (1) eliminating the data points that were below the values estimated by the linear regression and (2) performing a second linear regression through the remaining data points. This modified regression procedure is known as double regression (DR) [2]. However, DR has found limited use because its accuracy has not yet been validated [22].

By considering actual environmental conditions as zero flow criteria, Oishi et al. [23,24] defined a baseline upon ∆T_max_ values that were observed on days with particularly low vapor pressure deficit (VPD). Phillips et al. [13] also set their baseline on nights when VPD fell to zero for several hours. Regalado and Ritter [22] dynamically computed ∆T_max_ depending mainly on potential evapotranspiration, while Ward et al. [25] calculated their baseline from the relation between nocturnal sapflow estimates derived from the daily ∆T_max_ approach and nocturnal stomatal conductance that was simulated from data of whole-tree chamber experiments.

However, although various determination procedures are in use, little attention has been paid to assessing uncertainties related to the application of such alternative ∆T_max_ approaches. This study therefore aims to compare existing ∆T_max_ approaches and quantify their effects on sap flux density (g·m^−2^·s^−1^) estimations for mature Norway spruce trees in the Eifel National Park (Schleiden, Germany). Besides the described empirical ∆T_max_ approaches, we test the method of Oishi et al. [23,24] as a representative for VPD-based ∆T_max_ approaches and the ∆T_max_ simulation method of Regalado and Ritter [22]. For lack of whole tree chambers, the approach of Ward et al. [25] is not considered in this study. The outcome is analyzed on the sub-daily, daily and (intra-)seasonal scales.

Considering that the analyzed ∆T_max_ approaches have been designed to capture nocturnal sapflow where existing and that we only analyze days with unlikely nocturnal flow, the hypothesis is that all ∆T_max_ approaches yield the same sap flux density estimations as D. Deviations from D thus represent the uncertainty of sapflow computations, which is related to the ∆T_max_ determination approach.

## 2. Materials and Methods

### 2.1. Study Site

The study site (50°30′ N, 06°19′ E) is located in the 38 ha Wüstebach catchment (western Germany) where altitudes range from 595 m a.s.l. to 628 m a.s.l. [26]. Hillslopes are dominated by shallow Cambisols and Planosols while Gleysols and Histosols have developed in the groundwater-influenced riparian zone along the Wüstebach stream. The soils mainly show a silty clay loam texture with a medium to high coarse material fraction.

The climate is characterized by an annually mean temperature of 7 °C, a mean annual precipitation of 1100 to 1200 mm [27] and an average potential evapotranspiration of 630 mm [28]. The precipitation is more or less evenly distributed over the seasons with a slight peak in fall (~500 mm in contrast to ~300 mm during the other seasons). Thus, even in summer, periods of high transpirative power alternate with rainy days and respective low transpiration and sapflow activity. With 320 trees ha^−1^, the Wüstebach catchment is densely forested by Norway spruce (*Picea abies*). The trees were planted in 1949 [29] and have now reached a canopy height of ~25 m [28].

### 2.2. Sample Trees

The three sample trees are located at 50°30′18′′ N/6°19′52′′ E, 620 m a.s.l. at an ESE facing slope with a gradient of 8°. Mean diameter at breast height (DBH) is 54.8 cm, mean projected crown area (CA) amounts to 54.8 m^2^ and mean sapwood depth (SWD) is 5.7 cm, which was determined by drillhole analyses (Table 1). To ensure that our investigations are not overlaid by effects related to the individual phenological development of the trees, we used the overlap of the trees’ main growing periods as study period. The main growing period is defined as the time interval during which 5%–90% of the seasonal growth is reached [30] and could thus be determined on the basis of dendrometer data (cf. 2.4). The study period started on 25 May and ended on 14 August 2012.

### 2.3. Sapflow Measurements and Calculation

**Figure 1:**
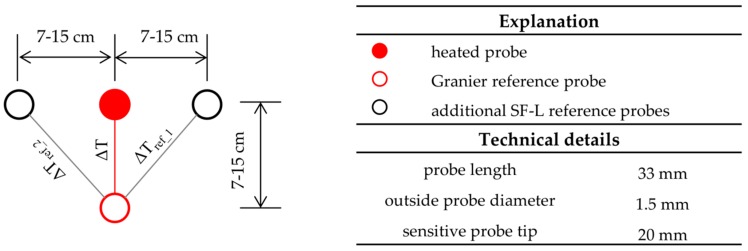
Installation scheme and technical details of the used Ecomatik sapflow sensors, type SF-L 20/33, according to [31]. The original ΔT between the heated probe and the Granier reference probe is corrected by substraction of the inner-wood temperature variations (ΔT_ref_1_, ΔT_ref_2_) recorded by the additional SF-L reference probes.

The improved Granier-type sapflow sensors that we used in our study (type SF-L 20/33, Ecomatik, Dachau, Germany) include an extra pair of thermocouples that are placed horizontally to the upper heated probe to account for natural innerwood temperature variations. For installation scheme and technical details of the sensors, see Figure 1. The mean of the inner-wood temperature variations recorded by the additional SF-L reference probes are subtracted from the values recorded by the classic Granier system before applying the Granier formula [31]. This pre-processing of the Granier sensor signal slightly affects absolute sapflow estimates. In this study, accounting for inner-wood temperature variations reduced mean seasonal sapflow by 3.1%. The sapflow sensors were installed in the outermost 3.3 cm of the sapwood on the north side of the sample trees at ~1.5 m above ground. We insulated our probes with reflective polystyrene and plastic boxes. Measured temperature gradients were recorded at a datalogger (type CR1000, Campbell Scientific Ltd., Logan, UT, USA) in 30-min intervals. Sap flux density (F_d_) was estimated in line with Granier [1,11].

Besides the classic Granier approach [1,11], we applied moving window approaches of different width (3, 5, 7 and 9 days), and the linear and double regression approaches [2]. Furthermore, we tested the application of one single ∆T_max_ (absolute maximum) and the physically-based methods of Oishi et al. [23,24] and Regalado and Ritter [22].

For the Oishi method, we applied the software *baseliner 4.beta* [24] which identifies ∆T_max_ when the following conditions are met: (1) nighttime; (2) stable ∆T; and (3) low VPD. We determined nighttime based on global radiation and set the radiation threshold for night-time definition 5.0 W·m^−2^. This value corresponds to the night-time definition of Daley and Phillips [12] that we also used for data selection (see below). Stable ∆T was identified when the coefficient of variation for a two-hour period was <0.01. Low VPD conditions were identified when mean VPD was less than 0.05 kPa for a two-hour period.

For the Regalado and Ritter approach, we applied the software FITDTMAX (available online: https://aritter.webs.ull.es/software_FITDTMAX.html) using the default transformed potential evapotranspiration (ETp*) limit of 0.1. Since our data had a temporal resolution of 30 min instead of the 15 min resolution used by Regalado and Ritter [22], we raised the proportionality tolerance from the default value of 0.05 to 0.1. To ensure the reliability of the modelled ∆T_max_ values and respective sap flux density estimates, we excluded days from our investigations where the coefficients of determination between the selected transformed potential evapotranspiration and ∆T were below 0.75 (for more details on the modeling procedure, see [22]).

To assess the impacts of the study period length and the position of the study period within the main growing period on ∆T_max_, we divided our study period into the following sub-periods: (1) 25 May to 22 June; (2) 23 June to 19 July; and (3) 19 July to 14 August. All ∆T_max_ approaches were applied to both the entire growing period and the sub-periods. Resulting sapflow densities were analyzed on the sub-daily (30 min resolution), daily and (intra-)seasonal scales. Although alternative ∆T_max_ approaches have been designed to capture nocturnal sapflux where existing and not to modify day-time sapflux densities, it is obvious that changes in ∆T_max_ affect both nocturnal and day-time F_d_ estimates [23]. To assess the magnitude of this effect, we separately investigated the effects of alternative ∆T_max_ approaches on nocturnal and day-time F_d_ estimates, respectively.

An overview of the applied ∆T_max_ approaches and their implementation and abbreviation are given in Table 2. All sap flux density estimations were evaluated with regard to (1) the results obtained by the original daily ∆T_max_ approach (D); (2) their applicability to data series of different length and (3) data plausibility in terms of climate feedback. Test statistics (one-sided Mann-Whitney U-tests) were applied to each data series.

To allow for taking D as the reference approach, days of potentially ongoing nocturnal sapflow were excluded from our investigations. We assumed nocturnal sapflow to potentially occur as a consequence of (1) nocturnal transpiration; (2) nocturnal tree growth or (3) nocturnal restoration of the tree’s internal water storage during periods of droughts [12,14].

Periods of ongoing nocturnal transpiration and tree growth were excluded by only using sapflow data from days on which nocturnal potential evapotranspiration fell to zero while at the same time relative stem extension (cf. 2.4) was zero or negative. Water limitations did not occur in our study period and could therefore be neglected as a driving factor for nocturnal water movements in the trees. Applying these criteria, from the original study period of 80 days, 76 days (Tree 1), 77 days (Tree 2) and 72 days (Tree 3) of unlikely nocturnal sapflow remained for investigation.

### 2.4. Environmental Measurements and Classification

Tree growth and diurnal stem extension were observed by point dendrometers (type DR, Ecomatik, Dachau, Germany). The relative stem extension was determined by taking the difference between two consecutive dendrometer measures. To monitor soil water content, we installed two SPADE sensor probes (SPADE, sceme.de GmbH, Horn-Bad Meinberg, Germany) in 5, 20 and 50 cm depths. Soil matric potential was recorded by an equitensiometer (EQ15, Ecomatik, Dachau, Germany) in 20 cm depth. To observe micro-climate on site, we measured air temperature and relative humidity at 2 m above soil surface (HygroClip2, Rotronic, Ettlingen, Germany). All data was recorded in a 30-min resolution (datalogger CR1000, Campbell Scientific).

For above-canopy meteorological investigations, we had access to half-hourly climate data (temperature, global radiation, relative humidity, potential evapotranspiration ET_pot_) of the TERENO Observation Network (weather station Schöneseiffen, 3.4 km east of the Wüstebach). Daily precipitation data was provided by the German Meteorological Service (DWD), weather station Kalterherberg (9.6 km west of the Wüstebach).

Actual evapotranspiration (ET_act_) was determined by an on-site eddy-covariance (EC) system installed at a height of about 38 m [32]. The tower is located in direct proximity to our sample trees within the Wüstebach catchment. Processed data was available in half-hourly resolution [28].

Based on the results of a pilot study in the same catchment, we divided the study period into days of distinct environmental conditions that were found to impact F_d_. These were (1) days of low/high global radiation, on which the daily global radiation was 0.5 of the standard deviations below/above the mean daily global radiation between May and September 2012 and (2) wet days, on which daily precipitation was above 5 mm and dry days, which were defined as the second day without precipitation. Days that met these conditions were analyzed separately. Nighttime was defined as the period during which radiation was less than 5.0 W·m^−2^ [12].

## 3. Results

### 3.1. Sub-Daily Scale

**Figure 2:**
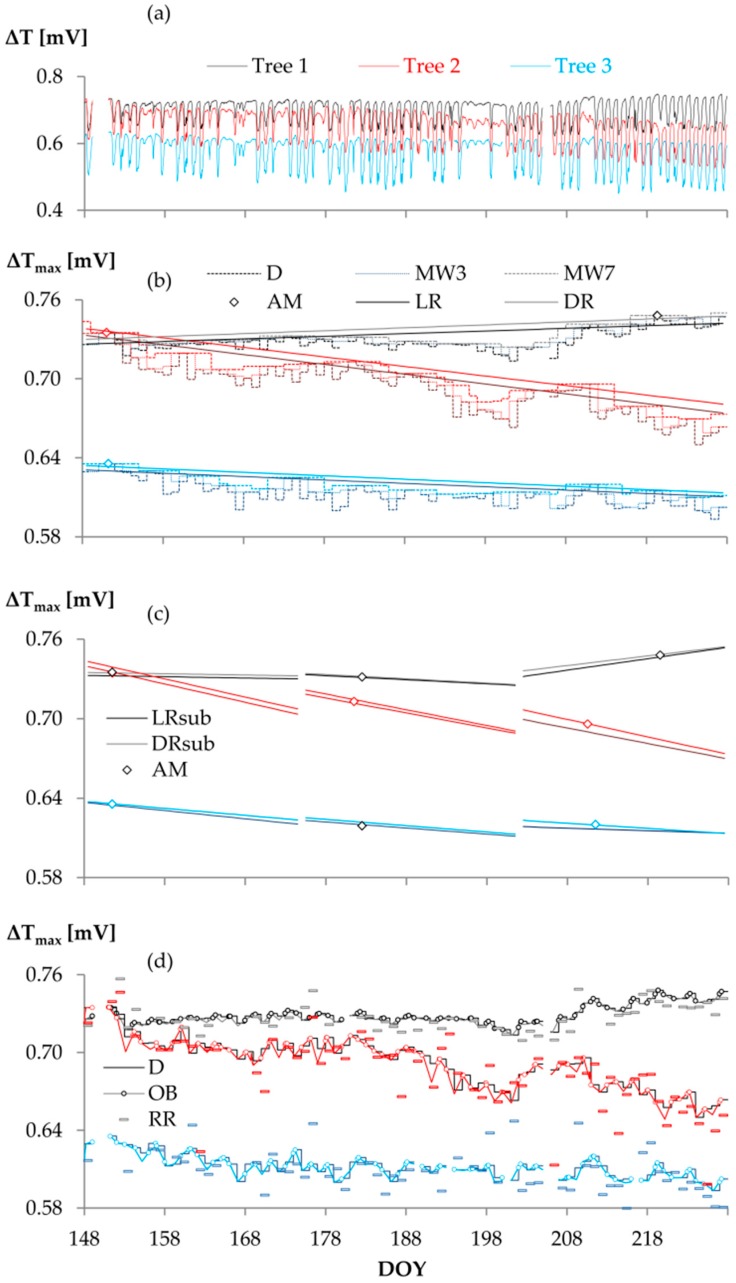
∆T and ∆T_max_ by determination approach and tree: (**a**) ∆T over growing season; (**b**) ∆T_max_ by moving window (MW3, MW7) and regression approaches applied to complete study period (LR, DR); (**c**) ∆T_max_ by regression approaches applied to sub-periods (LR_sub_, DR_sub_); (**d**) ∆T_max_ by physically-based approaches (OB, RR). Abbreviations of ∆T_max_ approaches according to Table 2.

**Figure 3:**
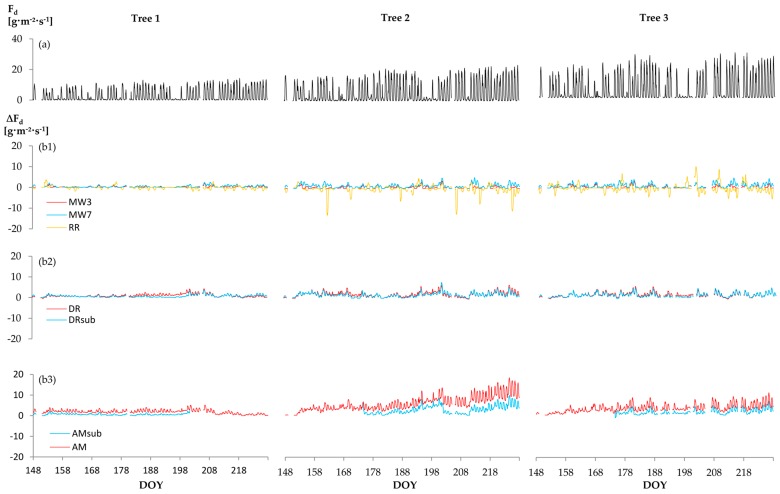
(**a**) Diurnal sap flux density by tree, calculated by the daily ∆T_max_ approach (F_d_) and (**b**) deviations (∆ F_d_) from D using those ∆T_max_ approaches that yielded the largest F_d_ differences among each other (**b1**): F_d_ deviations from D by MW3, MW7, RR; (**b2**): F_d_ deviations from D by DR applied to complete study period and sub-periods; (**b3**) F_d_ deviations from D by AM applied to complete study period and sub-periods). Abbreviations of ∆T_max_ approaches according to Table 2.

Maximum temperature gradients (∆T_max_) strongly vary depending on the ∆T_max_ approach (Figure 2). For the linear and double regressions, the reference period has a major impact on the resulting ∆T_max_ trend (Figure 2b,c). While the Oishi “baseliner” shows similar patterns as D, RR yields ∆T_max_ values that strongly vary about D (Figure 2d). All empirical ∆T_max_ approaches yielded higher mean sapflow densities (F_d_) than D (*p* < 0.01). F_d_ generally increased with increasing MW width; based on the test statistics (cf. 2.3), MW9 and LR were evaluated as equal (*p* > 0.1). The largest difference in sap flow density was computed between approaches RR < D < MW3 < MW7 < DR < AM (Figure 3).

Although the means of the sub-period LR distributions (hereinafter referred to as LR_sub_) still equaled the respective means of the MW9 distributions, the minority of LR_sub_ distributions were found to equal the respective parts of the LR distributions obtained from the complete study period ∆T_max_ regression. Some LR_sub_ distributions were significantly smaller and some significantly larger than the respective parts of LR. Likewise, we could not identify a distinct relation between DR_sub_ (sub-period DR) and other ∆T_max_ approaches.

Since ∆T and ∆T_max_ were found to develop dynamically over the growing season (Figure 2), the static AM approach led to significantly biased sapflow density distributions and was therefore not considered for further analysis.

From the physically-based approaches, both OB and RR yielded lower mean F_d_ than D (*p* < 0.01) when applied to the complete study period; applied to the sub-periods, the majority of the means of the OB distributions equaled those of the D distributions, while RR still yielded lower sapflow densities than D (*p* < 0.05, two exceptions). Comparing RR with OB, RR yielded either equal or lower mean F_d_ than OB. All tested ∆T_max_ approaches yielded higher nocturnal sapflow densities after radiation intensive days than after days of below-average radiation (*p* < 0.01).

**Figure 4:**
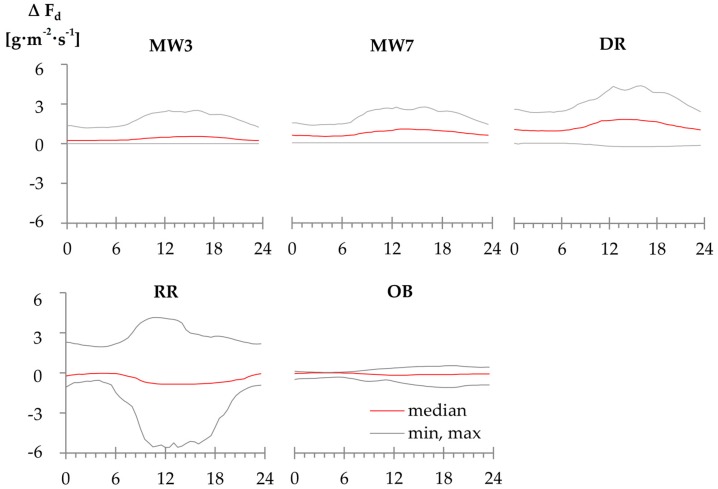
Average of the mean, minimum and maximum diurnal sap flux density deviations (∆ F_d_) from D using those ∆T_max_ approaches that yielded the largest F_d_ differences among each other. Abbreviations of ∆T_max_ approaches according to Table 2.

A comparison of empirical ∆T_max_ approaches and D showed that the averaged absolute day-time sap flux density increase was always higher than the mean F_d_ increase at nights (*p* < 0.01). For the physically-based approaches, in contrast, the difference to D during days and nights was either equal or higher during the nights than in the daytime (Figure 4).

Correlating F_d_ and climate variables (above-canopy vapor pressure deficit VPD_air_, below-canopy vapor pressure deficit VPD_stand_, radiation R; actual evapotranspiration ET_act_) all ∆T_max_ approaches yielded satisfactory coefficients of determination (R^2^ of 0.62 to 0.75).

### 3.2. Daily Scale

Over the entire study period, the physically based approaches and D were evaluated as equal. The empirical ∆T_max_ approaches, in contrast, led to mean daily sapflow densities that significantly differed from those calculated by the D approach (*p* = 0.05, one exception). Among the empirical ∆T_max_ approaches, we found a higher homogeneity on the daily scale than on the sub-daily scale. The most distinct variations were identified between approaches D, MW7, DR and AM. Sap flux density estimates by AM, however, significantly exceeded all alternative F_d_ estimations (*p* = 0.01) and led to a strongly biased increase of daily sapflow with progressing study period.

**Figure 5:**
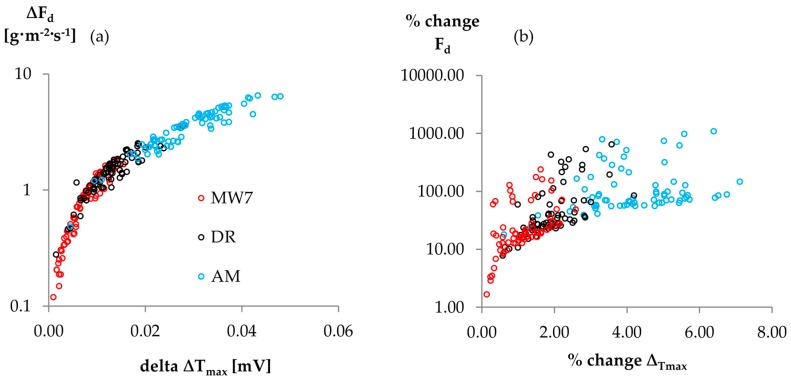
Mean (**a**) absolute variation and (**b**) percentage change of ΔT_max_ by approach and respective absolute variation and percentage change of mean daily sap flux density estimations. Deviations always with regard to the results obtained using the D approach. Abbreviation of ∆T_max_ approaches according to Table 2.

Figure 5 shows the absolute and relative mean daily F_d_ and ∆T_max_ increases for different empirical ∆T_max_ approaches. Neglecting the unreliable AM approach, the maximum absolute daily F_d_ increase was produced by DR, would correspond to a relative F_d_ increase of 106.0% and was induced by a ∆T_max_ increase of only 0.04 mV (5.52%).

**Figure 6:**
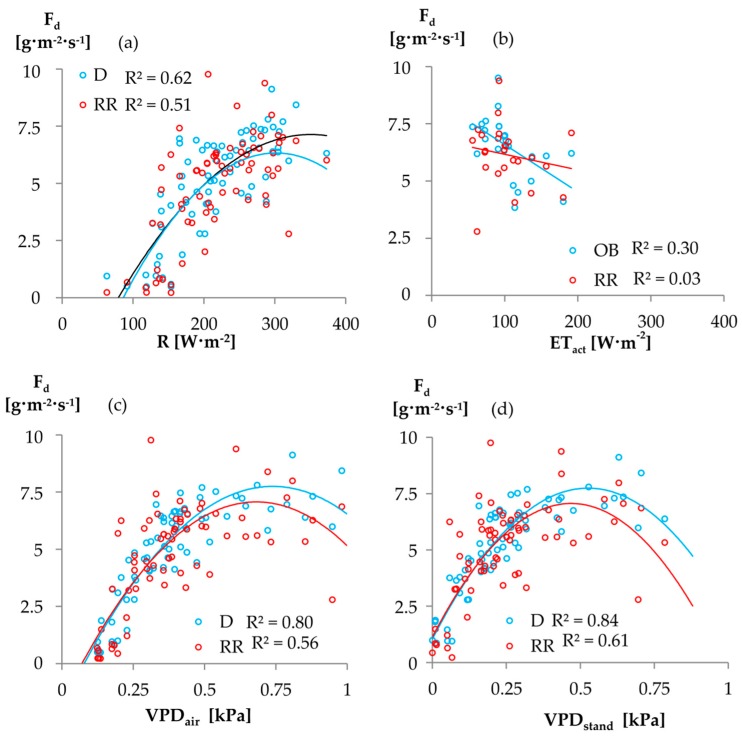
Relationship between climate variables and mean daily sap flux density F_d_ for different ΔT_max_ approaches (always best and worse correlation shown). (**a**) F_d_ correlation with global radiation R; (**b**) F_d_ correlation with actual evapotranspiration ET_act_ (correlation only shown for days of high radiation); (**c**) F_d_ correlation with vapor pressure deficit above canopy VPD_air_; (**d**) F_d_ correlation with on site vapor pressure deficit at 2 m above ground VPD_stand_. Abbreviations of ∆T_max_ approaches according to Table 2.

Analyzing the climate response among the ∆T_max_ approaches, we found well-matching relations between daily sapflow estimations, VPD_air_, VPD_stand_ and radiation (R^2^ of 0.51 to 0.84; Figure 6). Except for the poor ET_act_ correlations, the D approach showed the strongest climate feedbacks among the tested ∆T_max_ approaches. While the absolute difference of mean daily F_d_ estimations among the ∆T_max_ approaches was not related to any climate signal, for both MW7 and DR, the mean relative daily sap flux density increase was higher on low radiation days than on days of high radiation (*p* = 0.01).

### 3.3. (Intra-)Seasonal Scale

Dependent on ∆T_max_ approach and data series, mean (intra-)seasonal F_d_ varied from D by −13.9% (RR) to +137.6% (AM).

**Figure 7:**
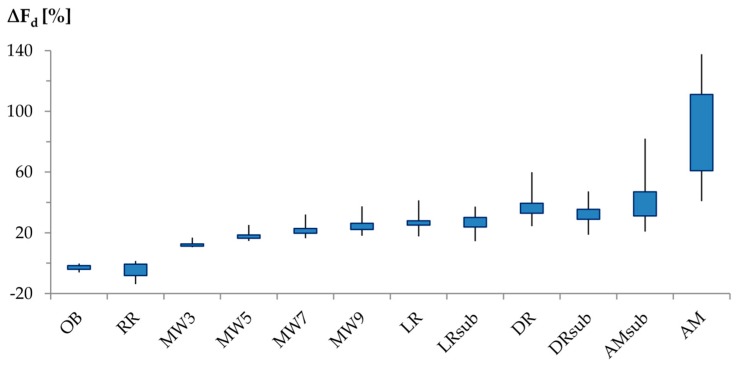
Percentage deviation of mean (intra-)seasonal sap flux density estimates using alternative ∆T_max_ approaches from estimates using daily ∆T_max_ (D). Abbreviations of ∆T_max_ approaches according to Table 2.

MW sapflow densities increased with increasing MW width, while F_d_ response on regression approaches strongly varied by time series and by the reference period they were applied to (Figure 7). Mean F_d_ derived from MW approaches was 12.5% (MW3) to 24.7% (MW9) higher than that derived from D, mean F_d_ calculated by regression approaches exceeded that of D by 26.0% (LR_sub_) to 38.5% (DR).

For those approaches that yielded equal results on the daily scale, mean discrepancies between mean (intra-)seasonal F_d_ did not exceed ±10%. However, discrepancies among the approaches strongly varied by time series, still reaching maximum variations of up to 24%.

Except from RR, all alternative ∆T_max_ approaches differed stronger from D on wet days of low radiation than on dry days of high radiation (Table 3).

## 4. Discussion

### 4.1. Sub-Daily Scale

We observed significant variations between most analyzed ∆T_max_ approaches. Among all ∆T_max_ approaches, the largest difference in sap flux density was computed between RR < D < MW3 < MW7 < DR < AM. Although absolute F_d_ deviations varied not only by the applied ∆T_max_ approach, but also by tree (Figure 3), mean percentage deviations were quite homogenous among the trees (Figure 7).

The variations between the MW approaches are particularly critical since MWs of different reference periods are often used in sap flow studies. Considering that we excluded days of likely nocturnal sapflux from our investigations, our results indicate that MW approaches produce a predictable amount of synthetic nighttime flow, and a more than proportional synthetic increase in daytime flux. The same applies for the regression approaches and AM with the limitation, however, that for LR, DR, and AM the outcome is not as predictable as for the MW approaches: As long as the same reference period was being used, we found no difference between sap flow densities calculated by LR and the MW approach LR was based on (MW9). However, LR sap flux density estimates strongly varied with the reference period length and its position within the vegetation period; being based on LR, the same applies for the DR approach. Another drawback of DR is that it does not show a constant relation to other ∆T_max_ approaches.

Not very surprisingly, the most biased sap flow density distributions were produced by AM: ∆T develops dynamically over the growing season and so should ∆T_max_ [23].

However, something we can pick from the analysis of AM and other empirical ∆T_max_ approaches is, that the uncertainty of F_d_ estimates increases with the number of days that lie between the captured ∆T_max_ values. Researchers should be aware of this problem, even when applying physically based ∆T_max_ approaches like OB, because particularly in environments, where zero-flow criteria are not met for a recognizable number of consecutive days, it might become a significant source of uncertainty. One solution to handle this uncertainty could be to define a maximum distance between the captured ∆T_max_ values. To define such a maximum distance, more research would be needed.

In our study, however, the potential problem of non-occurring zero flow criteria for OB sapflow estimations was of minor importance. We only analyzed days of anyway unlikely nocturnal flows and found that, depending on the study period, OB yielded either equal or lower sapflow densities than D. The sometimes slightly lower F_d_ estimations of OB result from the fact that some of the ∆T values that met the Oishi selection criteria were even lower than the daily maximum ∆T (Figure 2d). Thus, the outcome of OB does not only underline the plausibility of our data exclusion criteria, but also supports our hypothesis that MW and other empirical ∆T_max_ approaches produce kind of artificial day- and nighttime flows.

The finding that RR results in similar F_d_ estimations as OB indicates that the physically based approaches produce more consistent sapflow estimations than the empirical ∆T_max_ approaches do. Simulating ∆T_max_ from ∆T and micrometeorological variables, the RR approach has the advantage of not being affected by the potential problem of non-occurring zero flow criteria. However, yielding the weakest climate correlations among all tested ∆T_max_ approaches shows that RR has other drawbacks: The general fit between transformed ET_pot_ (ETp*) and sap flux density has great influence on the number of data points that are selected for ∆T_max_ determination. Thus, on days with differing ETp* and sapflow dynamics, it may happen that ∆T_max_ cannot be calculated due to a lack of fitting data points. In the study of Regalado and Ritter [22], this problem seems to having been of minor importance. However, Regalado and Ritter used data with a high temporal resolution of 15 min, so they always found enough data points that met their proportionality criteria. Although we raised the proportionality tolerance to a reasonable level for our 30 min data resolution, we had to exclude several days from our analysis, because the number of selected data points was not sufficient for a solid ∆T_max_ determination. Of course, to some extent, this kind of problem could have been handled by further adapting the proportionality tolerance and also the ETp* limit for night time definition. However, the higher we choose the proportionality tolerance for data selection, the lower turns the coefficient of determination between the selected ETp* and F_d_ points; and since ∆T_max_ is derived from the correlation between the selected data points, the reliability of the simulated ∆T_max_ values and respective F_d_ estimations would then decrease as well. Another drawback of the RR procedure is that the correlation strength between the selected ETp* and F_d_ values is strongly dependent on actual weather conditions: On wet days with low radiation we yielded mean R^2^ between selected ETp* and F_d_ of 0.72, while on dry days of high radiation mean R^2^ was 0.95, which is close to the R^2^ values reported by Regalado and Ritter [22]. We therefore conclude that on clear days without precipitation the RR approach may yield reliable ∆T_max_ and respective sapflow estimations, but should be handled with care, when unsteady weather conditions prevail.

Summarizing the above, it seems that for humid conditions without water limitations, D and OB lead to the most reliable sap flux density estimations among the ∆T_max_ approaches. For environments with potentially occurring nocturnal flows, OB might be the better choice, but more research is needed to verify the night-time flow detected by OB against a known standard. One of the main future challenges in this regard is, however, to create such a standard. So far, there is a lack of cost-efficient absolute reference measurements that enable us to detect real night-time flow and calibrate for it. Lundblad et al. [33] recalibrated the Granier formula against sapflow measurements of a tissue heat balance system [34]. However, the Čermák system also refers to a reference level of assumed zero flow conditions and is thus not solving the problem of nocturnal flow detection. Other studies use eddy covariance systems as an absolute reference for sapflow as one component of total ecosystem evapotranspiration [7,35]. However, EC systems have the disadvantage that they only capture total ecosystem fluxes and are known to measure imprecise at nights [36]. Consequently, it is neither possible to capture single tree transpiration with this method, nor does it make sense to correct nocturnal forest transpiration for EC system measurements. Ward et al. [25] conducted whole-tree chamber experiments to detect nocturnal transpiration and calibrate the Granier formula for it. They showed that accounting for real night-time flux is possible and matters. However, chamber experiments are expensive and difficult to implement. Particularly for adult trees and natural forest environments, a convincing solution for nocturnal flow assessment in Granier-type sapflow systems has not yet been found. Consequently, at this state, the most feasible options to deal with the problem of undetected night-time flow are (1) to accept the inability of Granier-type sapflow systems to detect ongoing nocturnal flux as a general constraint of the measuring approach (which holds the risk of underestimating sapflow and absolute sapflow rates matter in forest hydrological research) or (2) to apply physically-based approaches as the Oishi’s one including unknown uncertainty caused by the restrictions described above.

### 4.2. Daily Scale

Mean daily sapflow densities of the physically based approaches did not significantly differ from those of D. The results of the empirical ∆T_max_ approaches, in contrast, exceeded those of D by 9.8 (MW3) to 31.5% (DR).

This finding is in line with our findings on the sub-daily scale and indicates that the use of empirical ∆T_max_ approaches may become a significant source of uncertainty in daily sapflow estimations. For energy driven environments with unlikely nocturnal sapflow activity, our results suggest the application of D for daily sapflow estimations. D always showed the best correlation with the selected climate parameters, except from ET_act_ which was generally weak (cf. Figure 6). However, investigations by Wilson et al. [7] and Köstner [37] suggest that better correlations might have been achieved when data for soil evaporation and understory transpiration data had been available and subtracted from ET_act_ measured using an EC tower in advance. While OB yielded results comparable to D and might also be an option for environments with potentially occurring nocturnal flows (cf. 4.1), RR should only be applied with care: Although RR yielded absolute mean F_d_ estimates that did not significantly differ from that of D, it showed the weakest daily climate correlations among all ∆T_max_ approaches (also cf. 4.1).

### 4.3. (Intra-)Seasonal Scale

One important issue of forest hydrological research is the quantification of evapotranspiration and its components. Ringgaard et al. [8] reviewed that the individually determined evapotranspiration components in forests underestimate EC system measurements by up to 20%. There is broad evidence, that besides scaling issues and miscalculation of other evapotranspiration components, the processing of the raw sapflow signal is one of the main reasons for these discrepancies. However, our results show that the application of alternative ∆T_max_ approaches is not always the appropriate tool to address this problem. Empirical ∆T_max_ determination approaches translate any intermediate ∆T_max_ decrease into nocturnal flow activity, although the seasonal course of ∆T_max_ is also dependent on thermal wood properties and these may vary with tree water status and environmental conditions [2,5,38]. In our study, where conditions of unlikely nocturnal sapflow prevail, this mistranslation of the ∆T_max_ synthetically raised (intra-)seasonal sap flux density estimations of individual trees by between 10.5 (MW3), 57.8 (DR) and 137.6% (AM). In absolute values, (intra-)seasonal sap flux density estimates of the physically-based approaches were much more consistent and yielded similar results as D. However, applying the RR approach significantly decreased data plausibility on the sub-daily and daily scales. Consequently, OB was the only alternative ∆T_max_ approach that yielded convincing sap flux density estimations and has the potential to detect nocturnal flow, when occurring.

Nevertheless, more research is needed to validate detected night-time flows by absolute reference measurements. For this purpose, applicable measuring techniques are needed, that allow for absolute nocturnal flow detection. Another future challenge will be to deepen the understanding of the natural ∆T_max_ variability and to consider respective findings in the ∆T_max_ determination.

## 5. Conclusions

Based on the analyses of sapflow data of three spruce trees, we showed that the ∆T_max_ determination procedure has a major influence on Granier-based sap flux density estimations. While on days of unlikely nocturnal sapflow, physically-based ∆T_max_ determination approaches yield similar sap flux density estimations as the classic daily ∆T_max_ approach, other, empirical ∆T_max_ approaches produce synthetic flows that (1) significantly raise absolute sap flux density estimations on the sub-daily, daily and (intra-)seasonal scales; (2) affect sub-daily and daily sap flux density dynamics; and (3) reduce data plausibility in terms of climate feedbacks on the daily scale. We therefore conclude that the use of alternative ∆T_max_ approaches may be a significant source of uncertainty in sapflow estimations and complicates the comparability of sapflow studies.

For humid environments with unlikely nocturnal sapflow, our results suggest to apply the original daily ∆T_max_ determination or the physically-based OB approach. RR and other, empirical ∆T_max_ determination approaches were found to yield unsatisfactory results.

To improve Granier-type sapflow estimations, future research should focus more strongly on the development of applicable measuring approaches that allow for absolute nocturnal flow detection and respective recalibration of the Granier formula. Another future research focus should be the deepening of our understanding of the natural ∆T_max_ variability, which is related to wood properties and other eco-physiological parameters. Respective findings should be used to develop new ∆T_max_ approaches that allow for a solid, physically-based ∆T_max_ determination and for reliable absolute sap flux density computations.

## Figures and Tables

**Table 1:** Attributes of sample trees (DBH: diameter at breast height, SWD: sapwood depth, CA: projected crown area).

Tree	DBH (cm)	SWD (cm)	CA (m^2^)	Main Growing Period	
				Start	End
1	58.4	6.1	52.3	6 May	25 August
2	54.3	5.7	50.1	24 May	25 August
3	51.7	5.4	61.9	15 May	14 August
Means	54.8	5.7	54.8	25 May	14 August

**Table 2:** Theory and implementation of the applied ∆T_max_ approaches.

∆T_max_ Approach	ID	∆T_max_ Determination	References	Implementation in This Study
*Systematic approaches*				
Daily maximum	D	Daily maximum	[1]	Daily ∆T_max_ determination
Moving window	MW	Dynamic determination based on dynamic time windows of 3 (MW3) to 14 days (MW14)	[2,8,15,21]	Dynamic time windows of 3, 5, 7, 9 days, always starting 1, 2, 3, 4 days before the actual date of study
Linear regression	LR	First calculate local maxima of moving 10 day periods, then calculate new ∆T_max_ by LR of the local maxima and DOY	[2,11]	LR based on local maxima of 9 day period
Double regression	DR	Elimination of local ∆T_max_ below the LR line and new LR based on remaining data points	[2]	Regression and data point selection based on local maxima of 9 day period
Absolute maximum	AM	Absolute maximum within selected study period		Absolute maximum within selected study period
*Physically-based approaches*				
Oishi baseliner	OB	Identification of points in time where flow is likely zero, based on ∆T stability and biophysical conditions; baseline is set by interpolation between selected points; measured ∆T values that exceed the interpolation line are integrated into the baseline	[23,24]	Model setup for point selection: vapour pressure deficit threshold = 0.05 kPa; global radiation threshold for night-time definition = 5.0 W·m^−2^
Simulated ∆T_max_	RR	Daily simulation of ∆T_max_ based on the relationship between potential evapotranspiration and sapflow readings	[22]	Model setup: transformed potential evapotranspiration limit (ETp*) limit night-time definition = 0.1; proportionality tolerance = 0.1; exclusion of days with coefficients of determination between selected ETp* and sapflow readings < 0.75

**Table 3:** Percentage deviation of sapflow estimations yielded using alternative ∆T_max_ approaches from estimates using daily ∆T_max_ (D) for different climate conditions. Abbreviations of ∆T_max_ approaches according to Table 2.

∆T_max_ Approach	% Deviation from D	
	Dry Days, High Radiation	Wet Days, Low Radiation
OB	0	−10
RR	−13	3
MW3	7	25
MW5	12	36
MW7	17	45
MW9	20	53
LR	17	64
DR	24	95
AM	57	202
